# Empirical ways to identify novel Bedaquiline resistance mutations in AtpE

**DOI:** 10.1371/journal.pone.0217169

**Published:** 2019-05-29

**Authors:** Malancha Karmakar, Carlos H. M. Rodrigues, Kathryn E. Holt, Sarah J. Dunstan, Justin Denholm, David B. Ascher

**Affiliations:** 1 Victorian Tuberculosis Program, Melbourne Health, Victoria, Australia; 2 Department of Biochemistry and Molecular Biology, University of Melbourne, Melbourne, Victoria, Australia; 3 Department of Microbiology and Immunology, University of Melbourne, Melbourne, Victoria, Australia; 4 Structural Biology and Bioinformatics, Baker Heart and Diabetes Institute, Melbourne, Victoria, Australia; 5 The Peter Doherty Institute for Infection and Immunity, University of Melbourne, Victoria, Australia; 6 Department of Biochemistry, University of Cambridge, Cambridge, United Kingdom; St Petersburg Pasteur Institute, RUSSIAN FEDERATION

## Abstract

Clinical resistance against Bedaquiline, the first new anti-tuberculosis compound with a novel mechanism of action in over 40 years, has already been detected in *Mycobacterium tuberculosis*. As a new drug, however, there is currently insufficient clinical data to facilitate reliable and timely identification of genomic determinants of resistance. Here we investigate the structural basis for *M*. *tuberculosis* associated bedaquiline resistance in the drug target, AtpE. Together with the 9 previously identified resistance-associated variants in AtpE, 54 non-resistance-associated mutations were identified through comparisons of bedaquiline susceptibility across 23 different mycobacterial species. Computational analysis of the structural and functional consequences of these variants revealed that resistance associated variants were mainly localized at the drug binding site, disrupting key interactions with bedaquiline leading to reduced binding affinity. This was used to train a supervised predictive algorithm, which accurately identified likely resistance mutations (93.3% accuracy). Application of this model to circulating variants present in the Asia-Pacific region suggests that current circulating variants are likely to be susceptible to bedaquiline. We have made this model freely available through a user-friendly web interface called SUSPECT-BDQ, StrUctural Susceptibility PrEdiCTion for bedaquiline (http://biosig.unimelb.edu.au/suspect_bdq/). This tool could be useful for the rapid characterization of novel clinical variants, to help guide the effective use of bedaquiline, and to minimize the spread of clinical resistance.

## Introduction

Tuberculosis (TB) is the leading cause of infectious disease death worldwide, with over 10 million new cases and 1.6 million deaths in 2017 [1]. A disproportionate burden arises from the estimated 558,000 annual cases of rifampicin resistant TB (RR-TB) with 82% being multi-drug resistant (MDR), which is associated with lengthy, toxic therapy and high rates of mortality [1]. With limited therapeutic options available, especially for MDR-TB and extensively drug-resistant (XDR) TB, the introduction of new treatment options is urgently required. Bedaquiline, a new anti-TB drug with a novel mechanism of action, targeting the c-ring of ATP synthase (AtpE) [2], was approved for treatment for MDR-TB in 2012 [3, 4]. This innovative drug is potent against both actively replicating and dormant bacilli and has been shown to increase culture conversion in patients with MDR-TB [5]. The use of bedaquiline has expanded considerably in recent years, and has been recommended for more routine use in MDR-TB regimens [6], however clinical failures have already been observed [7, 8]. This necessitates a better understanding of how variants result in resistance to aid in the early detection of resistance.

Phenotypic, and increasingly genotypic, drug susceptibility testing (DST) is recognized as essential for effective individualization of TB therapy. However, while progress has been made in strengthening laboratory diagnostics, the TB community is still struggling to build up laboratory networks with the needed capacity for routine culture and DST [7, 9]. The World Health Organization (WHO) has strongly urged the development of accurate and reproducible DST for bedaquiline and recommended that in the absence of specific DST, bedaquiline resistance should be monitored through MIC assessment [10] with resistance development evaluated in patients with treatment failure or relapse. Early characterization of drug resistance mutations would assist TB patient management and avoid treating individuals with ineffective toxic regimens [11, 12], but capacity for rapid genotypic prediction of bedaquiline resistance is limited by the identification of few known resistance associated variants [13].

In an era of rapidly expanding use of molecular technologies, including whole genome sequencing, tools for evaluating the impact of novel mutations are increasingly vital, particularly for drug resistance to novel and emerging medications such as bedaquiline. Though culture-based detection of resistance will remain the gold standard, *in silico* analyses can support informed decision-making. We have previously shown that the analysis of how variants can affect protein structure and function can be used to reliably characterize how variants lead to drug resistance [14–18]. Using this approach, we have shown that drug resistant mutations can be rapidly, accurately and pre-emptively predicted, guiding drug development [19–22] and clinical diagnosis [23].

*In-vitro* selection [24] and clinical studies [25] have shown that variants in the *atpE* gene can lead to bedaquiline resistance. To support rapid identification of potential bedaquiline resistance mutations, we considered whether structural information of the drug target could help guide clinical inference on genomic variants. Using a suite of well-established computational tools for characterizing the molecular consequences of mutations on protein structure and function, we have assessed the effects of mutations on the biophysical changes of AtpE folding, stability and on drug binding affinity. This was used to characterize how mutations in AtpE lead to resistance, and to train a predictive multilayer perceptron (feedforward artificial neural network) algorithm to characterize novel AtpE variants.

## Methods

### Data sets

Resistant variants from *in-vitro* selection studies were curated [13, 24, 26] along with a natural variant [4, 27] and used for model development. Susceptible variants were identified using a novel homology approach, where the genomes of all mycobacteria species sensitive to the drug [28] were aligned, therefore inferring that any present variants were likely to be susceptible. Clinically observed bedaquiline resistant *atpE* variants were curated from published reports [25]. The Vietnam dataset consists of whole genome sequences of 1635 *Mycobacterium tuberculosis* (*Mtb*) strains isolated from patients with pulmonary TB in Ho Chi Minh City, Vietnam. The *Mtb* genome data is available in NCBI BioProject [ID: PRJNA355614; http://www.ncbi.nlm.nih.gov/bioproject/355614]. Details of the clinical study and the whole genome dataset are found in Thai et al [29] and Holt et al [15].

### Homology modeling of AtpE

The structure of *Mtb* AtpE was modelled with MODELLER [30] using the experimental crystal structure of *Mycobacterium phlei* (*M*. *phlei*) AtpE (PDB ID: 4V1F). The model was then minimized in Prime and bedaquiline docked into the apo structure using Glide (Schrӧdinger Suite).

### Modelling the biophysical consequences of missense variants

The structural consequences of the AtpE polymorphisms were assessed to account for all the potential effects of the mutations. The effects of mutations on protein folding and stability were assessed using SDM [31], mCSM-Stability [32] and DUET [33], and their effects on protein flexibility and conformation was predicted using normal mode analysis by DynaMut [34]. The effect of the difference on the protein-protein interactions between the protomers of AtpE were predicted using mCSM-PPI [32]. The effect of the changes on the binding affinity of bedaquiline towards AtpE were predicted using mCSM-Lig [35–37]. These approaches are novel machine-learning algorithms that use graph-based signatures to represent the structural and chemical environment of the wild-type 3D structure of a protein to quantitatively predict the effects of point mutations. Additionally, SNAP2 [38] was used to provide additional evolutionary based information.

### Machine learning

To build the binary classifier, a multilayer perceptron neural network algorithm was trained, based on the implementation available through the Weka toolkit [39]. The resistant variants were up-sampled to create a more balanced model [40]. The training dataset constituted of 50 non-resistant associated variants and 5 resistant associated variants, while the blind test dataset constituted of 4 non-resistant associated variants and 4 resistant associated variants. To avoid over-biasing, the train and blind test dataset were non-redundant with respect to residue position. The model was trained and evaluated using jackknife [41] and leave-one-residue-position-out validation. The classification model was evaluated based on metrics, including the Area Under the ROC curve (AUC), precision and accuracy. Statistical analysis was performed using RStudio (version 3.1.1).

### Webserver development

The server front-end was built using materialize CSS framework version 1.0.0, while the back-end was built in Python via the Flask framework (version 0.12.2). It is hosted on a Linux server running Apache.

## Results

**Fig 1:**
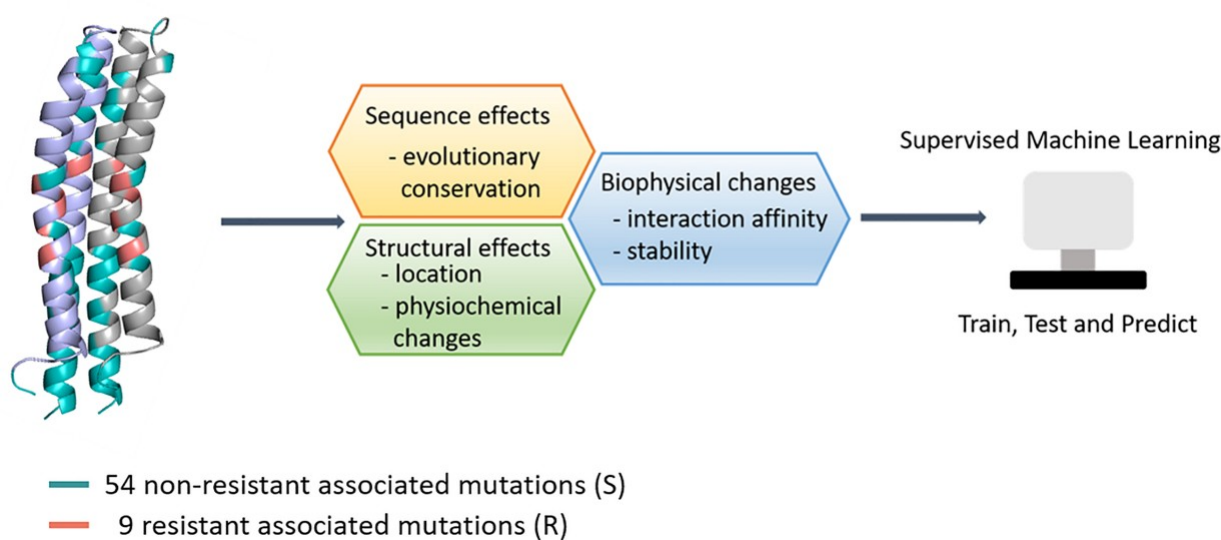
Methodology. This workflow highlights important steps in the methodology and how the main components of the algorithms are computed. In our analysis we used 54 non-resistant associated mutations and 9 resistant mutations for the biophysical analysis, followed by training and validation of our empirical model using a supervised machine learning algorithm.

We used a structure-guided approach to understand the protein structure of the drug target AtpE and machine learning to build an empirical tool that could identify likely resistant mutations. The pipeline used to analyze the variants and train a multilayer perceptron neural network algorithm is shown in Fig 1.

### Structural information: The drug binding domain

**Fig 2:**
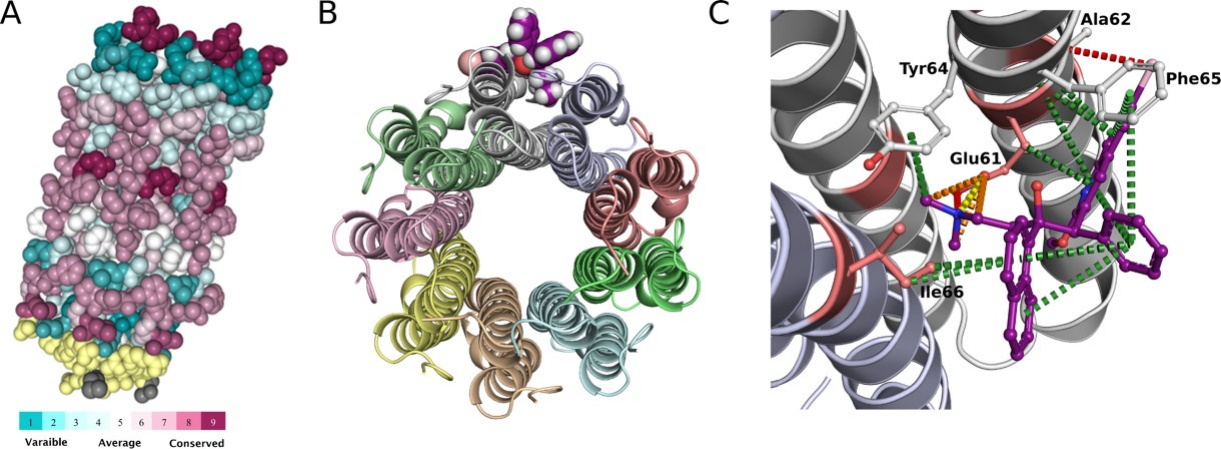
Structure and sequence information. (A) ConSurf analysis of AtpE (*M*. *tuberculosis*) where the evolutionary rates of conservation are color-coded on to the structure. (B) The experimental crystal structure of AtpE bound to Bedaquiline (purple). (C) The key molecular interaction between Bedaquiline (ball and stick representation; purple) and AtpE: ionic bond (yellow), π-interactions (green), proximal hydrogen bond (red) and weak polar van der Waal clashes (orange). The known resistance mutations are shown as salmon red (sticks) on the cartoon representation of the AtpE structure.

A homology model of *Mtb* H37Rv AtpE was built using the existing experimental crystal structure of AtpE from *Mycobacterium phlei* (PDB ID: 4V1F) [42], which shares a high sequence identity with the *Mtb* protein (84.9%). The protomer model was an alpha helical hairpin structure comprising two membrane-spanning helices connected by a hydrophilic loop. The homo-oligomeric construct was built using the *M*. *phlei* structure as a guide, as the *Mtb* protein has been previously shown to assemble as a homo-nonamer [43] (Fig 2A and 2B). The cylindrical palisade model contained an internal hydrophobic cavity where phospholipid had been proposed to bind. The conserved proton binding residue (E61) was located sandwiched between adjacent protomers and equidistantly distributed along the center of the hydrophobic membrane bilayer.

The top docking poses of bedaquiline with the nonamer homology model identified a pose consistent with that observed in the *M*. *phlei* structure. The drug binding cleft was located at the interface of two protomers, with amino acid residues E61, A62, Y64, F65 from one protomer and I66 from the adjacent protomer defining the drug binding cleft. Analysis of the molecular interactions with Arpeggio [44] highlighted a strong network of polar interactions between the drug and AtpE (Fig 2C). Of particular interest, the diethylaminomethyl group of bedaquiline specifically interacted with the conserved proton binding residue E61, making tight ionic and hydrogen bonds with the carboxyl group of E61 (S1 Fig). In the docked model, bedaquiline also made strong π-interactions with residues Y64 and I66, and a hydrogen bond to A62.

### Variant calling

**Fig 3:**
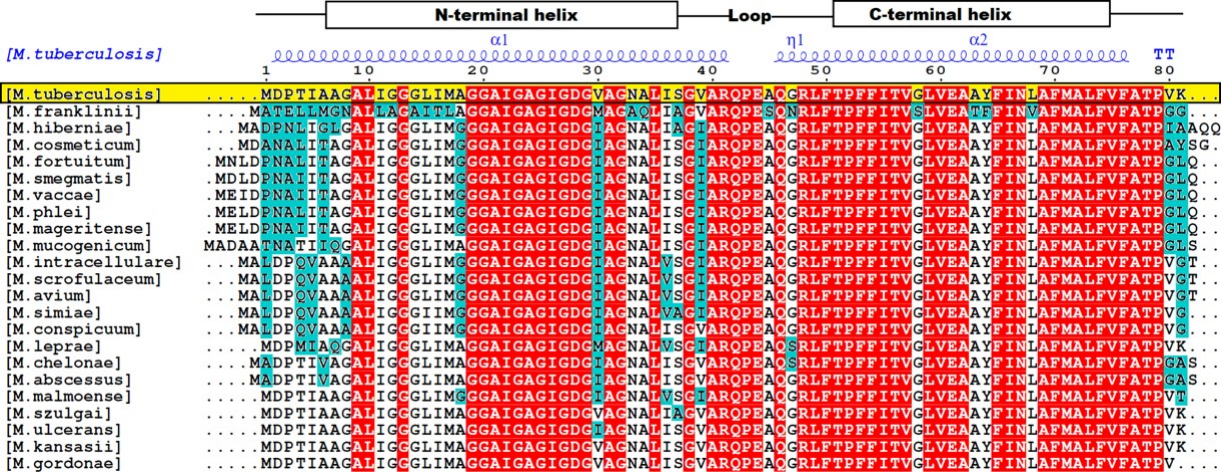
Non-resistant associated variant assignment. This image highlights the sequence alignment of 23 mycobacterial species sensitive to Bedaquiline. Residues that were different to the reference *M*.*tuberculosis* sequence (in yellow) are highlighted in teal, and were chosen as non-resistant associated variants for building the empirical model. The conserved residues are shown in red. The secondary structure of the AtpE protein is shown above the sequences in blue (α = alpha helix, η = loop). This image was created using ESPript 3 [56].

We identified 9 previously published bedaquiline resistant non-synonymous single nucleotide variants (nsSNVs) from *in-vitro* selection experiments [4, 13, 24, 26]. To identify AtpE mutations not associated with drug resistance, we examined sequence variation amongst AtpE sequences from 23 mycobacterial species that have been shown to be phenotypically sensitive to the drug [27, 45–49] (Fig 3). Due to the high degree of sequence conservation across mycobacterial AtpE sequences (~ 66% sequence homology; Clustal Omega), variations between strains shown to be susceptible to bedaquiline were inferred to not be associated with drug resistance. Through comparison against the *Mtb* sequence (highlighted in yellow in Fig 3), 54 non-resistance-associated variants were identified (shown in teal in Fig 3).

Understanding the structural basis of resistance is important to facilitate the rapid identification of novel resistance variants, aiding efforts to minimize the rapid development of resistance [23]. The 54 non-resistance-associated variants (“S”) and 9 resistant variants (“R”) were mapped on the protein structure of AtpE (Fig 1). Most of the non-resistance-associated mutations were located on the N-terminal surface exposed inner loop of AtpE. Conserved regions (highlighted red in Fig 3) were evident, mainly on the C-terminal or the outer loop and embedded in the lipid bilayer of the membrane. All resistance-associated mutations were localized within 5 Å of the known drug binding site, which we refer to as the “resistance hotspot”.

### Structural and biophysical consequences of AtpE variants

The resistant associated variants were all predicted by SNAP2 [38] to be more functionally deleterious than the non-resistance associated variants, reflecting the resistant associated variants are in a more conserved region of the protein. In order to better understand the molecular consequences of the mutations on AtpE structure and function, the mutations were analyzed in the context of both the apo and complexed protomeric structures. The impact of resistant and non-resistant associated mutations on protein folding, stability and conformation were assessed using SDM [31], mCSM-Stability [32], DUET [33] and DynaMut [34]. The effect of the variants on the affinity of the protomers to form the cylindrical palisade homo-oligomer were examined using mCSM-PPI [32], and the effect of the variants on the binding affinity for bedaquiline were assessed using mCSM-Lig [37].

**Fig 4:**
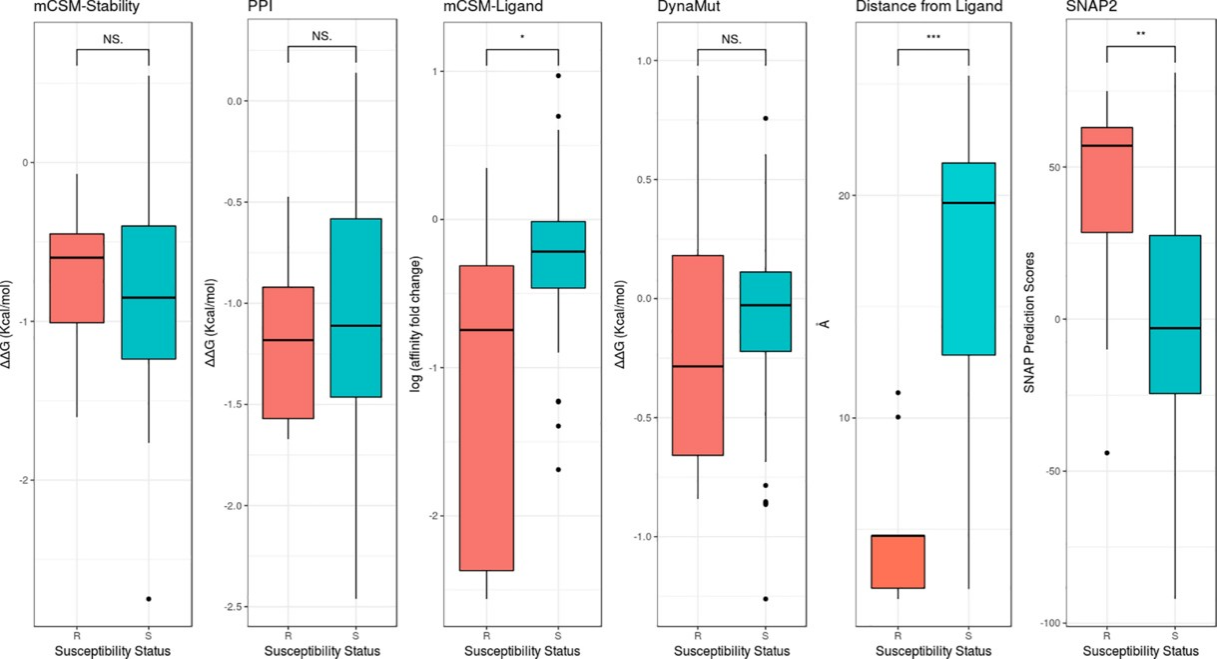
PCA analysis. Boxplot representation of all the features used to build the predictive model. The resistant associated mutations (R) are represented as red and the non-resistant associated mutations (S) as teal. (* p<0.05, ** p<0.005, *** p<0.0001, NS p>0.5 by Welch two sample t-test).

Analysis of the variant effects on protomer stability and the formation of the cylindrical palisade did not reveal statistically significant differences between resistant and non-resistance-associated variants (Fig 4). This is consistent with recent work that showed in order to minimize fitness costs, resistant associated variants in drug targets tended to have mild effects on protein stability [50]. The largest destabilizing effect observed amongst the resistance-associated variants using mCSM-Stability and DUET was for the conservative mutation E61D (ΔΔG = -1.1 Kcal/mol), however normal mode analysis by DynaMut suggested that the E61D mutation would not destabilize the structure and was only associated with mild conformational changes (S1 Fig). Examination of residue conservation across 150 homologous sequences using ConSurf [51] showed the equivalent residue position in many species was an Asp, suggesting its introduction is unlikely to have a large structural or functional effect.

While all nine resistant variants were within 5 Å of the ligands, five in particular, A63M, A63P, E61D, L59V and I66M, were within 2.5 Å and making direct interactions with bedaquiline. Modelling of these mutations revealed that most of them would result in complete loss of these intermolecular interactions (S2 Fig). For example, E61 upon mutation to Asp would result in loss of these strong ionic and hydrogen bonds with bedaquiline. Interestingly, the mutation of I66 to Met and L59 to Val mutation revealed the formation of new interactions, although the overall binding affinity was predicted to be lower by CSM-lig. Most of the non-resistant associated variants were located distal to the bedaquiline binding site.

Analysis of predicted changes in bedaquiline binding affinity upon mutation using mCSM-Lig revealed a significant difference between variants associated with resistance or not associated with resistance (Fig 4). The non-resistance associated variants were associated with mild mCSM-Lig predicted changes in bedaquiline binding affinity (average of -0.25 log affinity fold change). This would be consistent with the mutations leading to minimal change in, or even increasing, drug binding affinity. The average predicted log fold change in binding affinity obtained for the 9 resistant mutations, by contrast, was -1.29 log affinity fold change, indicating that they would likely disrupt bedaquiline binding. Among them, all four D28 resistant variants were predicted to the largest destabilising effect on bedaquiline binding (-2.5 log affinity fold change on average). D28 is positioned on the inner helix of the protomer and is 4.7 Å from the drug binding site. When D28 was substituted with either Ala or Gly, a loss in inter-helical interactions and a gain in flexibility was observed, and when substituted to Pro and Val it led to a gain in intra-molecular interactions and rigidification of the AtpE structures (S2 Fig).

### Machine learning algorithm: Multilayer perceptron network

Building on this structural analysis, we tested whether these structural features could be used to train a supervised machine learning algorithm capable of accurately predicting resistant associated variants. To avoid over-training, the 54 non-resistant and 9 resistant variants were split into a training and blind test dataset. Our training dataset constituted of 50 non-resistant associated variants and 5 resistant associated variants (A63V, A63P, I66M, L59V, E61D). Due to the small sample size, to balance the dataset, the resistant variants in the training dataset were oversampled (duplicated). The remaining 4 resistant (all D28 mutations) and 4 non-resistant associated (I11L, L15T, A34Q and A45S) variants in the blind test were positioned non-redundant with those in the training.

**Fig 5:**
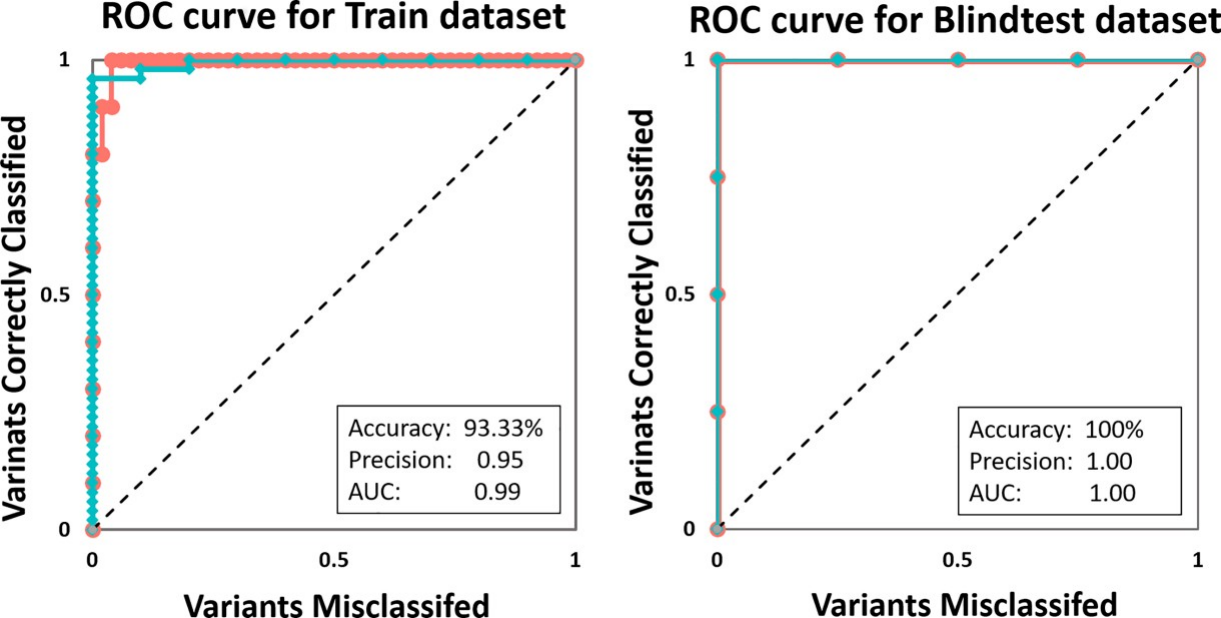
Evaluation metric. The ROC curve shows that using the structural and functional consequences of the variants, we were able to accurately identify resistant (red) and non-resistant associated (teal) variants.

A list of features tested in method development is described in S1 Table. As discussed above, the features that best distinguished between the classes include distance from ligand binding site (“Distance from Ligand”, p < 0.0001), mCSM-Lig (p = 0.026) and SNAP2 (p < 0.0001) (Fig 4). Using jackknife and leave-one-residue-position-out validation, models trained using multilayer perceptron neural networks yielded the strongest balanced performance. The final model correctly classified 93.33% and 100% of variants in the training and blind test datasets respectively (Fig 5, Table 1). The comparative performance across iterative non-redundant blind datasets suggested that the model was not over-fitted.

**Table 1:** Evaluation metrics of the train and blind test dataset.

Multilayer Perceptron (MLP)	Precision score	Recall	F-measure	ROC area	PRC area
Train Dataset	0.952	0.933	0.938	0.970	0.967
Blind test Dataset	1.000	1.000	1.000	1.000	1.000

The classifier revealed that variants with mild effects on protein stability and conformation (DynaMut < 0.28 Kcal/mol and DUET < -1.65 Kcal/mol), located close to the docked bedaquiline (distance from ligand < 6.36 Å) were likely to be associated with resistance. A closer examination of the four incorrectly classified non-resistant associated variant in the train dataset revealed that three of them, G58S, A63T and L68V, were positioned very close to the bedaquiline binding site (< 2.5 Å) and N33A had a large predicted change in binding affinity (-1.4 log affinity fold change); indicating that these mutations might have direct consequences on bedaquiline binding.

### Clinically identified resistance associated variants

Using a model trained without the D28 variants, we analyzed the recently reported clinical *atpE* bedaquiline resistant variants [25]. Both D28N and A63V were both predicted by the model to lead to bedaquiline resistance, consistent with the clinical data. Looking at these mutations within the structure, the mutation at D28 would disrupt interactions made by the wild-type residue to bedaquiline, consistent with the mCSM-Lig predictions that it would lead to a significant reduction in ligand binding affinity (S3 Fig; -1.87 log affinity fold change). Interestingly, while A63 did not make interactions directly with bedaquiline, the mutation to Val would lead to steric clashes with the bound ligand and prevent bedaquiline binding (S3 Fig).

### Vietnam data analysis

We also used this approach to predict the sensitivity of two *atpE* nsSNVs, I16V and P52L, identified through whole genomic sequencing of *Mtb* strains isolated from 1635 TB patients in Vietnam [15]. The predictive tool classified the reported nsSNPs to be non-resistant associated variants. These variants were located approximately 10 Å away from the bedaquiline binding site, and mutations at these residues were not predicted to disrupt any interactions with bedaquiline (S4 Fig). As these samples had been collected from patients that had not been administered bedaquiline, it provided confidence that in our large analysis of patients in Vietnam there were no circulating strains likely to be resistant to bedaquiline.

### SUSPECT-BDQ webserver

We have implemented SUSPECT-BDQ as a user-friendly, freely available web server http://biosig.unimelb.edu.au/suspect_bdq/. SUSPECT-BDQ provides two different input options. The “Single Mutation” option allows users to predict whether a mutation will be characterized as either Resistant or Susceptible. For this option, the server requires the point mutation to be specified as a text string containing the wild-type residue one-letter code, its corresponding position on the structure and the mutant one-letter code. The “Mutation List” option allows the user to upload a file with a list of mutations in a file for batch processing. In order to assist users to submit their mutations for analysis, sample submission entries are available for both input options and a help page is also available via the top navigation bar.

For the “Single Mutation” option, the web server displays the prediction outcome of SUSPECT-BDQ alongside with details of the user input data, information on the residue environment and parameters used on the prediction (S5 Fig). In addition, an interactive 3D viewer, built using NGL [52] allows for analysis of non-covalent inter-residue interactions for the position specified in the input calculated with Arpeggio [44] for wild-type and mutant structures. For the “Mutation List” option, the results are summarized in a downloadable table from which users can access details for each single mutation. A 3D viewer is also shown and each wild-type residue from the input list is colored according to the predicted effect.

## Discussion

Early genomic detection of resistance is crucial for tailoring individual therapy and preventing the onward transmission of resistant infection. This is especially of importance to limit the spread of resistance to bedaquiline, one of the few treatment options for XDR-TB. While significant progress has been made in terms of innovative tools to understand and quantify the different range of effects in which a mutation or a set of mutations can give rise to a drug-resistant phenotype, a gap still exists when integrating these predictions and drawing conclusions regarding causality and the strength of associations observed. This is compounded by the need for detailed information regarding the system/protein. The availability of scalable, effective computational methods to assess mutational effects creates new opportunities for developing integrated approaches and deciphering complex genomic background patterns, shedding light on their role in the emergence of a given phenotype and molecular mechanisms of action [19].

Here we have used a computational approach to better understand the molecular mechanism of drug resistance within the context of the protein’s 3D structure. A machine learning algorithm was used to build a predictive tool which could pre-emptively determine novel bedaquiline resistant mutations within *atpE*. We began our investigation by studying the interaction dynamics between the c-ring of ATP synthase bound to bedaquiline. The correlations of conformational changes and Gibb’s free energy provided novel molecular insights into how resistance variants affected bedaquiline binding but led to minimal disruption of protein folding and dynamics. Mapping of all the mutations on the crystal structure helped us identify the “mutational hotspot” for AtpE, which was in proximity to the drug binding site. We saw that resistance associated variants were more likely to be located within this resistance hotspot, and lead to a significant disruption in bedaquiline binding. Interestingly, the characterized resistant variants did not lead to large changes in protein folding, stability or oligomeric state, which would impose a larger fitness penalty [50].

This *in silico* biophysical information was used to build a predictive algorithm that accurately identified resistant mutations. We then prepared a comprehensive mutational dataset that contained the predictions of all possible mutations in AtpE, which we have made available through a web-based interface: SUSPECT_BDQ (http://biosig.unimelb.edu.au/suspect_bdq/). These analyses highlight the power of considering the structural environment of a mutation to understand the molecular and biological consequences [53]. As a relatively novel drug, there is still a paucity of reliable information regarding resistance mutations. While limited by the relatively small available datasets, repeated stratified non-redundant blind testing revealed the model was very robust. This associative approach thus helped us establish a set of guidelines which adds to the missing information in the database for new TB drugs like bedaquiline. It also provides a molecular understanding of how variants in AtpE affect ligand binding, leading to resistance, providing insight to guide development of second-generation inhibitors.

We intend further development of this tool through expanded genomic targets, and evaluation using additional clinical isolates. In particular we intend to extend SUSPECT_BDQ to include non-target based resistance to bedaquiline, which has been linked to mutations in *Rv0678* [54], a transcriptional repressor of the gene encoding the MmpS5-MmpL5 efflux pump, and *pepQ* (*Rv2535c*) [55], a putative Xaa-Pro aminopeptidase. Both are associated with low-level of resistance and therefore we did not include them in the study. However, low level resistance may have clinical significance in some settings, and future work will further evaluate other potentially important loci. Additionally, testing this tool on further clinical isolates will enhance the efficiency of the tool to predict the consequences of novel mutations.

## Conclusion

This novel computational approach can enhance the impact of genome sequencing in identifying and characterizing variants more accurately and may therefore assist in guiding optimal usage of bedaquiline. The results obtained from our empirical tool is promising and should help facilitate routine genotypic drug susceptibility testing for bedaquiline and stimulate further research to help avoid the emergence of resistance to this new treatment through early detection.

## Supporting information

S1 Table The list of different features used to build the empirical model for predicting novel resistance associated mutations in bedaquiline. (PDF)

S1 Fig Detailed molecular interactions between the key proton binding residue E61, and upon its mutation to Asp, with bedaquiline. The wild-type residue is shown in cyan and mutant in salmon red in ball and stick representation. Bedaquiline is shown in purple (ball and stick representation). Hydrogen bonds are shown as orange dashes and ionic bond in yellow.
(TIF)

S2 Fig Images of intermolecular interactions made by the wild-type residue (shown as cyan) and the mutant amino acid (shown as salmon red). Hydrogen bonds are shown in red, halogen bonds in blue, ionic bonds in yellow, hydrophobic bonds in green, π bonds in grey.
(TIF)

S3 Fig Detailed molecular interactions between two clinically observed bedaquiline resistant variants, with the drug. The wild type residue is shown in cyan and mutant in salmon red in ball and stick representation. Bedaquiline is shown in purple (ball and stick representation). Halogen bonds are represented in blue dashes (amide-amide interaction) and π-bond as grey dashes.
(TIF)

S4 Fig The localization of two circulating *atpE* variants relative to the bedaquiline binding pocket. The wild type residues are shown in cyan and mutant in salmon red in ball and stick representation. Bedaquiline is shown in purple (ball and stick representation).
(TIF)

S5 Fig SUSPECT-BDQ webserver. Web-server results page for a single point mutation prediction. The predicted outcome is shown alongside with complementary information on the submitted mutation. An interactive 3D viewer allows for analysis of non-covalent interactions for both the wild type and mutant residue. In both cases controllers are provided in order to hide or show specific interactions and customize molecule representation.
(TIF)
