# Supplementary material for: Empirical ways to identify novel Bedaquiline resistance mutations in AtpE
Source: PLoS One. 2019 May 29;14(5):e0217169. doi: 10.1371/journal.pone.0217169 (PMC6541270; doi:10.1371/journal.pone.0217169)
Supplement: S1 Table — (PDF) [file pone.0217169.s001.pdf]

S1 Table: The list of different features used to build the empirical model for predicting novel resistance associated mutations in bedaquiline.

**Feature**	**Effect measured**	**Technique**	**p-value***
mCSM_Stability	Protein stability	Graph based signatures	0.31
SDM	Protein stability	Graph based signatures	0.90
DUET	Protein stability	Graph based signatures	0.65
mCSM_PPI	Protein-protein interaction	Graph based signatures	0.68
DynaMut	Conformational flexibility	Normal mode analysis	0.60
ΔΔG ENCoM	Conformational flexibility	Normal mode analysis	0.66
ΔΔS ENCoM	Changes in Entropy	Normal mode analysis	0.66
mCSM_Lig	Ligand binding affinity	Graph based signatures	0.03
Distance from ligand binding site	Distance of the mutation from the drug (bedaquiline) binding site	Perl script (in-house)	< 2.2e-16
SNAP2	Effect of single nucleotide substitution	Neural Network	0.0002

*Welch two sample t-test
