# Supplementary figures and images for: Empirical ways to identify novel Bedaquiline resistance mutations in AtpE
Source: PLoS One. 2019 May 29;14(5):e0217169. doi: 10.1371/journal.pone.0217169 (PMC6541270; doi:10.1371/journal.pone.0217169)

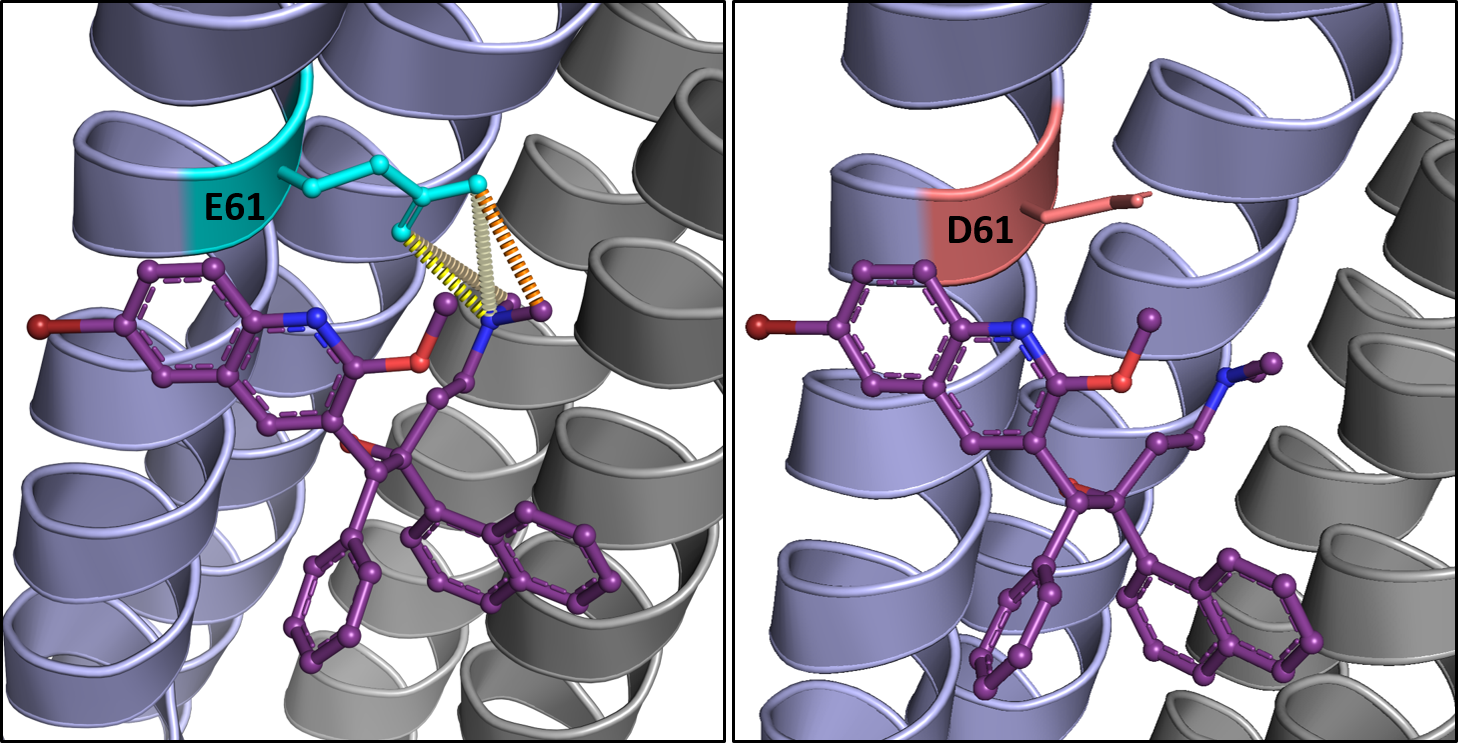

Supplement: S1 Fig — The wild-type residue is shown in cyan and mutant in salmon red in ball and stick representation. Bedaquiline is shown in purple (ball and stick representation). Hydrogen bonds are shown as orange dashes and ionic bond in yellow. (TIF) [file pone.0217169.s002.tif]

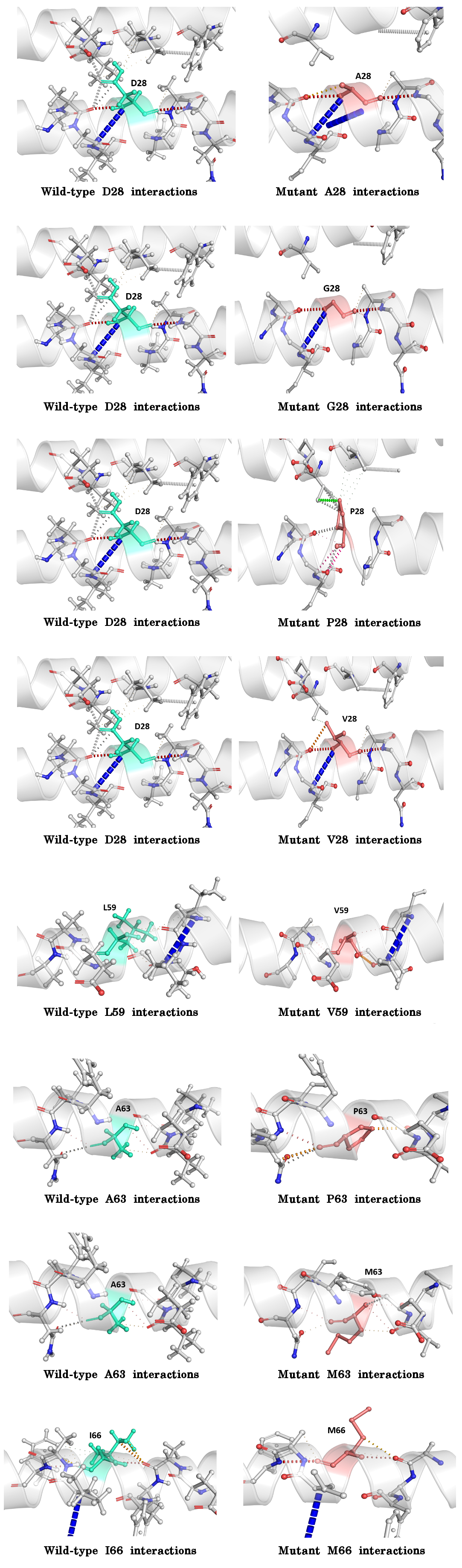

Supplement: S2 Fig — Hydrogen bonds are shown in red, halogen bonds in blue, ionic bonds in yellow, hydrophobic bonds in green, π bonds in grey. (TIF) [file pone.0217169.s003.tif]

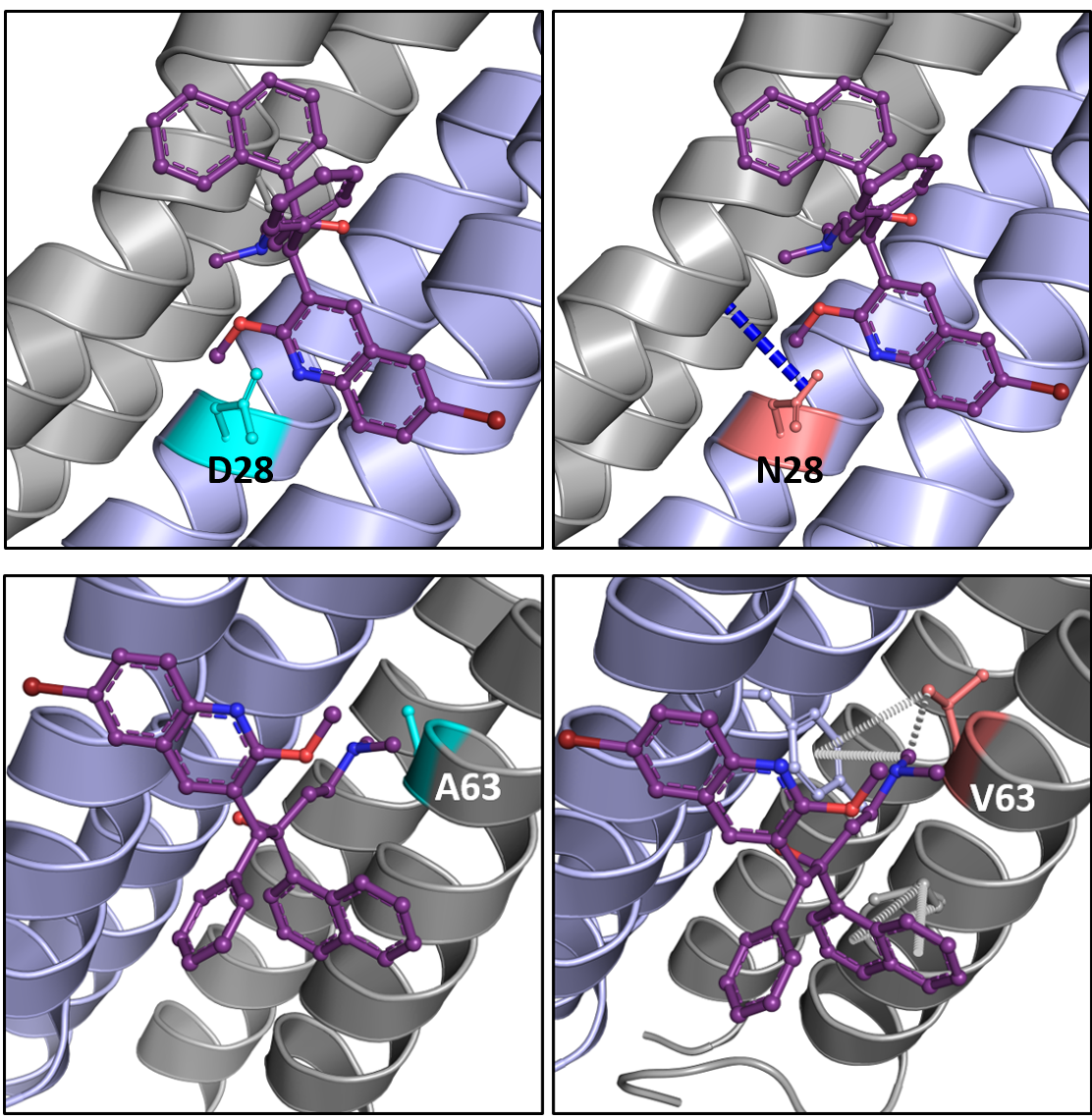

Supplement: S3 Fig — The wild type residue is shown in cyan and mutant in salmon red in ball and stick representation. Bedaquiline is shown in purple (ball and stick representation). Halogen bonds are represented in blue dashes (amide-amide interaction) and π-bond as grey dashes. (TIF) [file pone.0217169.s004.tif]

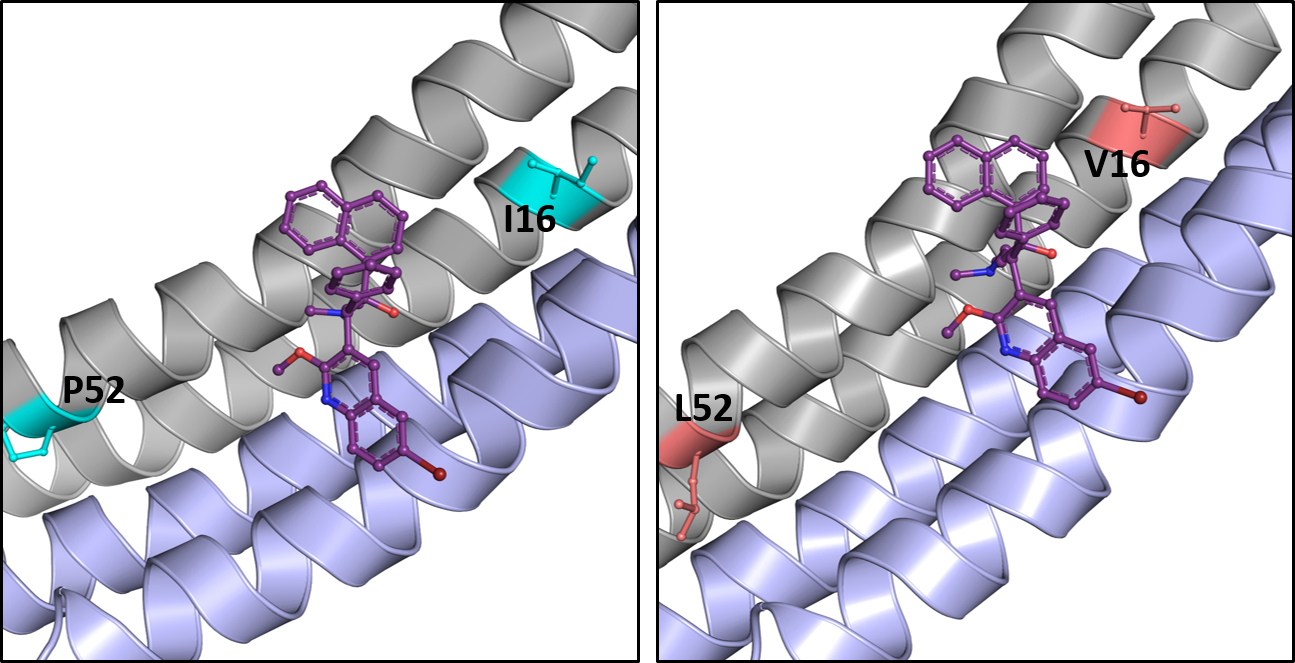

Supplement: S4 Fig — The wild type residues are shown in cyan and mutant in salmon red in ball and stick representation. Bedaquiline is shown in purple (ball and stick representation). (TIF) [file pone.0217169.s005.tif]

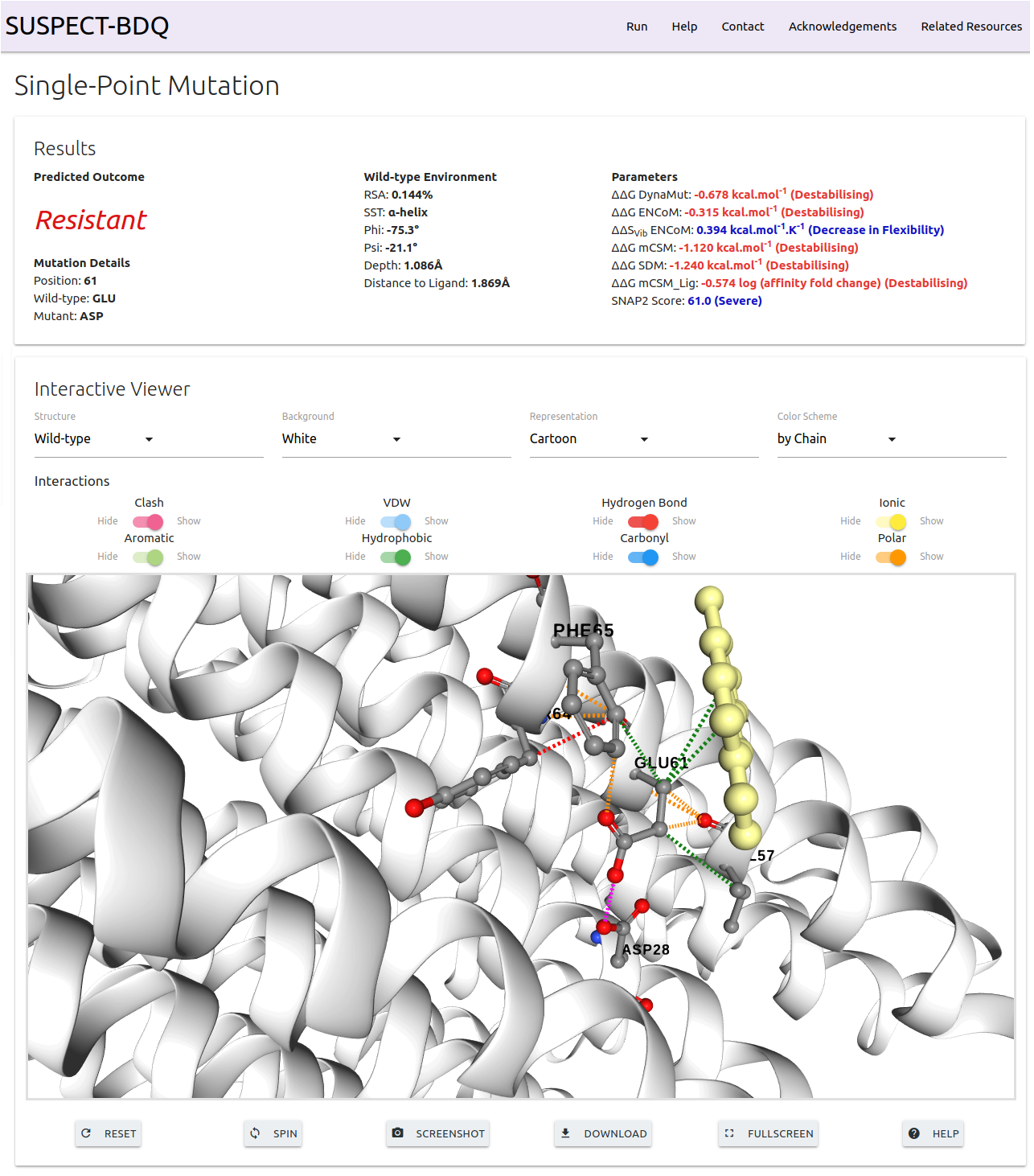

Supplement: S5 Fig — Web-server results page for a single point mutation prediction. The predicted outcome is shown alongside with complementary information on the submitted mutation. An interactive 3D viewer allows for analysis of non-covalent interactions for both the wild type and mutant residue. In both cases controllers are provided in order to hide or show specific interactions and customize molecule representation. (TIF) [file pone.0217169.s006.tif]
